# Bioactive Compounds, Antioxidant Activity, and Biological Effects of European Cranberry (*Vaccinium oxycoccos*)

**DOI:** 10.3390/molecules24010024

**Published:** 2018-12-21

**Authors:** Tunde Jurikova, Sona Skrovankova, Jiri Mlcek, Stefan Balla, Lukas Snopek

**Affiliations:** 1 Institute for teacher training, Faculty of Central European Studies, Constantine the Philosopher University in Nitra, SK-949 74 Nitra, Slovakia; tjurikova@ukf.sk (T.J.); sballa@ukf.sk (S.B.); 2 Department of Food Analysis and Chemistry, Faculty of Technology, Tomas Bata University in Zlín, CZ-760 01 Zlín, Czech Republic; skrovankova@utb.cz (S.S.); lsnopek@utb.cz (L.S.)

**Keywords:** cranberry, *Vaccinium oxycoccos*, polyphenols, antioxidant effect, biological activities

## Abstract

Lesser known fruits or underutilized fruit species are recently of great research interest due to the presence of phytochemicals that manifest many biological effects. European cranberry, *Vaccinium oxycoccos* fruit, as an important representative of this group, is a valuable source of antioxidants and other biologically active substances, similar to American cranberry (*V. macrocarpon*) which is well known and studied. European cranberry fruit is rich especially in polyphenolic compounds anthocyanins (12.4–207.3 mg/100 g fw), proanthocyanins (1.5–5.3 mg/100 g fw), and flavonols, especially quercetin (0.52–15.4 mg/100 g fw), which mostly contribute to the antioxidant activity of the fruit. Small cranberry is also important due to its various biological effects such as urinary tract protection (proanthocyanidins), antibacterial and antifungal properties (quercetin, proanthocyanidins, anthocyanins), cardioprotective (proanthocyanidins) and anticancer activities (proanthocyanidins), and utilization in food (juice drinks, jams, jellies, sauces, additive to meat products) and pharmacological industries, and in folk medicine.

## 1. Introduction

Berries, especially members of several families such as *Ericaceae*, belong to the best dietary sources of bioactive compounds. They have a typical flavor and often possess antioxidant properties, and therefore, are of great interest for nutritionists and food technologists [1]. The genus *Vaccinium* of family *Ericaceae* comprises more than 450 species across Europe, North America, Central America, Central and South East Africa, Madagascar, Japan, and Asia [2]. Blueberry (*Vaccinium ashei*, *V. angustifolium* Aiton, *V. corymbosum* L.), bilberry (*Vaccinuim myrtillus*), cranberry (*Vaccinium macrocarpon, V. oxycoccos*), huckleberry (*Vaccinium ovatum, V. parvifolium*), and lingonberry (*Vaccinium vitis-idaea*) are the most known and popular berries of this genus.

Lots of researchers have focused their attention on “large cranberry” or “American cranberry” (*Vaccinium macrocarpon* Aits) which is native in the northeastern part of the USA and widely commercially planted in British Columbia, Canada. Also lingonberry or cowberry or “rock cranberry*” (Vaccinium vitis-idaea*), native in North America and Europe, is the considerable lesser known crop. But till now only little research has dealt with “European cranberry” (*Vaccinium oxycoccos* L.), commonly known as “small cranberry” or “bog cranberry” [3,4]. *Vaccinium oxyccocos* plants include forms such as the little-leaf cranberry, *V. oxycoccos f. microphylla,* syn. = *Oxycoccus microcarpos* (Turcz.), and the larger-leaf form *V. oxycoccos L. subsp. paulustris* = *Oxycoccus quadripetalus* (Turcz.) [5]. However, according to Côté et al. [6] the taxonomic relationship between the cytotypes is uncertain, and nowadays *Vaccinium oxycoccos* is considered as a complex of diploid and polyploid plants.

In comparison with the large cranberry, the geographical distribution of European cranberry is considerably wider. It occurs in forest areas in Europe, Asia, and North America. The species of this shrub are widely commercially cultivated in Russia and Estonia, and also Lithuania [7]. It is an evergreen shrub with creeping stems that grows on peat in low drained sites. In European conditions it usually appears on sphagnum bogs in the north-western part of the European continent as far as North Asia and Japan. Cranberries ripen during late August and through September and can persist on plants until spring. The berries have pink, red or dark red color, strong acidic flavor and can be pear- or egg-shaped, round, oval, oblate or cylindrical [4,7,8].

Generally, the bioactive compounds in different types of berries contain mainly phenolic compounds such as flavonoids and ascorbic acid. These compounds, either individually or combined, are responsible for various health benefits of berries [1]. While the biologically active substances of large cranberry have been relatively extensively studied, there are only few studies focused on European small cranberry (*V. oxycoccos*). Similarly, also health effects of *V. macrocarpon* in prevention of some chronic diseases are well examined, wild cranberry fruits, including *V. oxycoccos*, did not get enough attention yet, even the fruit is widely used in food and pharmaceutical industries. It is great for use in juice drinks, jams, jellies, and sauces, and also as an additive to meat products [9]. European cranberry fruits represent an important natural source of antioxidants, such as polyphenolic compounds (i.e., anthocyanins, flavonols, phenolic acids, and proanthocyanidins), and ascorbic acid that are all attributed to antioxidant properties [10,11].

The review offers a recent view of European cranberry as an underutilized berry crop in respect to biologically active substances, especially polyphenolic compounds, antioxidants, antioxidant activity of berries, and different biological activities.

## 2. Bioactive Compounds of European Cranberry

Nowadays, the interest in bioactive compounds of European cranberry has increased due to its long-standing usage in folk medicine, especially in Eastern Europe, Finland, Sweden, and Russia [12]. On the other hand, there are only a few papers presenting results of studies relating to the bioactive compounds profile of the fruit.

According to Brown et al. [3] there are approximately 8000–10,000 phytochemicals detected in *V. macrocarpon*, *V. vitis-idaea*, and *V. oxycoccos.* The berries of large cranberry and European cranberry can be characterized by the accumulation of a high level of phenolic compounds, such as anthocyanins, flavonoids, and phenolic acids [8]. The phenolic compounds are important for plants for their normal growth and defense against biological and environmental stresses, infection, and injury [13]. Generally, cranberries have a diverse phytochemical profile with phenolic acids such as hydroxycinnamic acid, three classes of flavonoids (i.e., flavonols, anthocyanins, and proanthocyanidins), catechins, and triterpenoids [14].

Similarly to American cranberry, European cranberry contains flavonoids, anthocyanins, and other bioactive compounds with antioxidant activity and also a great amount of organic acids, and vitamin C as well [15]. Česonienė et al. [16] compared the amount of biologically active compounds among 40 genotypes (13 certified cultivars and 27 wild clones) of *V. oxycoccos* fruit of different origins (Estonian, Russian, and Lithuanian), grown under uniform ecological conditions in Lithuania. They found great variation in anthocyanin content, organic acids, and sugar content in fruits of cultivated types and wild clones, therefore the content of presented compounds differs depending on the cultivars. Analogously to the berries of *V. macrocarpon*, *V. oxycoccos* berries also contain citric acid (10.8 to 54.3 g/kg), malic (14.1 to 43.3 g/kg), and quinic (3.81 to 13.3 g/kg) acids as the main organic acids. The average content of fructose in fruits (42.1 g/kg) was analogous to glucose content (45.1 g/kg). The HPLC (high-performance liquid chromatography) method with UV and MS (mass spectrometry) detection used Jensen et al. [17] for the quantification of hydrophilic organic acids of cranberry, lingonberry, and blueberry juices. The highest content of hydrophilic carboxylic acids was evaluated for cranberries (2.67–3.57%) and lingonberries (2.27–3.05%), and a much lower amount was in blueberries (0.35–0.75%), with the presence of quinic acid, malic, shikimic, and citric acids.

Also ascorbic acid (vitamin C) is accumulated in cranberries. Due to some researchers European varieties have lower content of ascorbic acid than American cranberries; however, the results for vitamin C amounts in these fruits are quite dissimilar [18]. Tikuma et al. [19] mentioned that fresh berries of wild cranberry *V. oxycoccos* contain more ascorbic acid (31 mg/100 g) in comparison to cultivars of large cranberry (cultivars “Early Black”, “Stevens”, “Bergman”, “Pilgrim”). Especially the Latvian bred big cranberry cultivar “Septembra” showed a higher amount of ascorbic acid compared to other surveyed species. As Viskelis et al. [20] reported, the amount of ascorbic acid in American berries increases during ripening, from the beginning of ripening with white berries to 50% reddish berries, and ripe berries on average from 9.25 mg/100 g to 14.2 mg/100 g, and slightly decreases in overripe berries (10.3 mg/100 g).

Cranberry fruit represents an exceptional source of bioactive compounds of which also fatty acids have high biological activity even though the lipid amount in berries is low. The lipid profile of different berries reflects their taxonomy. There is a high amount of C18 unsaturated fatty acids in fresh berries, and also phytosterols, as it have been proved by GC-MS analyses [21]. The chemical composition of wild cranberry (*V. oxycoccos*) extracts from fresh fruit originating from the Russian Siberia, growing under natural conditions, and analyzed by GC-MS showed as major constituents benzyl alcohol, α-terpineol and 2-methylbutyric acid, and malic, citric, benzoic, and cinnamic acids in addition to fatty alcohols and acids [22].

### 2.1. Phenolic Compounds of European Cranberry

The berries of European cranberry belong to important sources of phenolic compounds, similarly as other berries. The phenolic content among different distinguished berry genera, such as family *Ericaceae* with genus *Vaccinium*; family *Rosaceae* with genera *Rubus*, *Fragaria*, *Sorbus*, *Aronia*; family *Grossulariaceae* with genus *Ribes*; and family *Empetraceae* with genus *Empetrum*, varies considerably as Moyer et al. [23] and Kähkönen et al. [24] found out. They evaluated anthocyanins as the main phenolic constituents in cranberries, in bilberries too, but not in lingonberries, belonging also to the genus *Vaccinium*, where flavanols and procyanidins predominate.

Generally, cranberry fruit is characterized by a diverse phytochemical profile with flavonoids such as flavonols, anthocyanins, and proanthocyanidins; catechins, phenolic acids, and triterpenoids.

The overview of major phenolic compounds in European cranberry (*V. oxycoccos*) is shown in Table 1.

Borowska et al. [31] provided the comparative study on polyphenols of wild-grown common cranberry (*Vaccinium oxycoccus*) and American cranberry cultivars “Ben Lear”, “Bergman”, “Early Richard”, “Pilgrim”, and “Stevens”, all originating from Poland. Statistically significant differences (*p* < 0.05) were found for total polyphenols and anthocyanins in the fruit of the analyzed cultivars. Total phenolic contents for American cultivars were in the range of 192.1 (“Pilgrim”) to 374.2 mg/100 g (“Ben Lear”), European cranberry cultivar reached 288.5 mg/100 g. The fruit of common cranberry contained the highest quantity of trans-resveratrol (712.3 mg/g), large cranberry ranged from 533.4 (“Stevens”) to 598.2 mg/g (“Ben Lear”). Tikuma et al. [19] also found in cultivars of *V. macrocarpon* Ait. much more phenolics than in European cranberry in the previous study. In the cultivar “Early Black” there was determined the highest amount of phenolics (441 mg/100 g) in comparison to other cultivars (“Stevens”, “Bergman”, “Pilgrim”), whereas the results showed significant differences in biochemical composition between the studied cranberry cultivars and species.

The content of total phenolics among clones of European cranberry cultivated in Lithuania ascertained Česonienė et al. [27]. Cranberry fruits of 21 clones, in different shape and berry size raised under the same growth conditions, accumulated from 224.1 mg/100 g to 498.2 mg/100 g of phenolic compounds. However, the relationship between the total amount of phenolics and berry weight of *Vaccinium oxycoccos* was only weak with a regression coefficient of R^2^ = 0.22. Negative correlation between the average berry weight and total phenolics content was detected by Česonienė et al. [8]. Anthocyanins in cranberry clones comprised 18.3% to 42.7% of total phenolic content [27]. The quantification of total polyphenols confirmed variations in their content depending mainly on the studied cultivars. Also Česonienė et al. [28] demonstrated in their study that the biochemical components of *V. macrocarpon* and *V. oxycoccos* juices are affected by genotype.

Results of the study of Povilaityté et al. [18] have shown that there are differences in the total amount of phenolics among American cranberry and European cranberry cultivars too. The berries of European cultivars accumulated from 100.4 mg/100 g (“Virussare”) to 154.8 mg/100 g (“Soontagana”), whereas American cultivars had about twice to four times higher content (192.3–676.4 mg/100 g).

Eighteen clones of European cranberry of Lithuanian origin from strictly protected areas Čepkeliai and Žuvintas were tested by Česonienė et al. [8] for the amount of total phenolics. Clones of *Vaccinium oxycoccos* accumulated different levels of phenolic compounds. The phenolic content ranged from 197 to 584 mg/100 g. The amount of phenolics in Čepkeliai clones was assessed on average 389 mg/100 g, in Žuvintas clones 347 mg/100 g. Except for genotype, the content of phenolics was also dependent on place of samples origin.

Both large cranberry (*Vaccinium macrocarpon*) as a commercially used crop and European cranberry (*Vaccinium oxycoccos*) as a traditionally used crop accumulated a high level of polyphenols. The comparison of the polyphenolic spectrum of *Vaccinium oxycoccos* and *Vaccinium macrocarpon* in fruit and pomace extract (crushed cranberry macerated with the solvent and its filtrate is concentrated by solvent evaporation) is shown in Table 2. Researchers [11,32] assessed more phenolic acids and flavonols in European cranberry pomace extracts than in fruit extracts, and the presence of resveratrol in pomace extract that was not found in fruit extract. The results of the studies also pointed out that fruits of European cranberry represent more valuable sources of caffeic acid and quercetin with higher values of total flavonols in comparison to American cranberry (*Vaccinium macrocarpon*).

Polyphenolic profiles of cranberries were studied also by Koponen et al. [29], Ogawa et al. [30], and Määttä-Riihinen et al. [33]. They determined that the concentration of hydroxycinnamic acids in European cranberry represented 7.6 mg/100 g, anthocyanins content in the berries was in the range of 66–86 mg/100 g, flavonols 27 mg/100 g, flavan-3-ols 3.1 mg/100 g, and proanthocyanidins were in the amount of 1.5–2.0 mg/100 g.

Researches of Häkkinen and Törrönen [34] and Viskelis et al. [20] have shown that berries of European cranberry grown in colder climates, without fertilizers or pesticides, can be characterized by higher content of phenolics than the cultivars grown in a milder climate. The differences in an accumulation of phenolic compounds can be also given by various conditions of cultivation, region, weather conditions, harvesting time, and maturity stage.

Kivimäki et al. [26] analyzed phenolic composition of cold-compressed berry juice from European cranberry. The results of the experiment showed that the content of total phenolic compounds in cranberry juice reached up to 29.4 mg/100 g which was less than in lingonberry or blackcurrant juices, as it reached about ½ or one third of their values. As for particular phenolic components the juice contained anthocyanins in the amount 12.4 mg/100 g, hydroxycinammic acids 5.2 mg/100 g, g flavan-3-ols 0.7 mg/100 g, flavonols 5.7 mg/100 g, and proanthocyanidins in the content of 5.3 mg/100 g.

The study of Mazur and Borowska [35] showed that the phenolic amount in the cranberry (*V. oxycoccos*) products is dependent on the used technological processes. Thus, in frozen fruits the content of phenolics is much lower (178.5 mg gallic acid equivalent (GAE) /100 g) than in freeze-dried fruits (678.9 mg GAE/100 g). Lyophilization of the fruits of this species resulted in the phenolic content reduction compared to fresh fruit.

Kylli et al. [36] compared the phenolic spectrum of European cranberry and lingonberry fruits. The major phenolic fraction of cranberry and lingonberry presents flavan-3-ols and proanthocyanidins. The main phenolic compounds in both fruits were proanthocyanidins, representing 63% and 71% of the total phenolic compounds. Anthocyanins (16% and 15%), flavonols (14% and 9%), and hydroxycinnamic acid (7% and 5%) were also detected. In cranberries there were therefore more anthocyanins, flavanols, and hydroxycinnamic acids than in lingonberries.

Ehala et al. [15] summed up that the main phenolic compounds identified in cranberry were quercetin and *trans*-resveratrol. These results are in agreement with the study by Taruscio et al. [37]. Resveratrol is an important antioxidant, phytoalexin stilbenoid, (3,5,4’-trihydroxy-*trans*-stilbene) that is found in high content in fruits such as cowberry (*Vaccinium vitis-idae*) (3.0 mg/100 g fw), followed by European cranberry (1.9 mg/100 g) and red currant (1.7 mg/100 g), and then bilberry and strawberry [15].

Also, ursolic acid (3β-hydroxy-urs-12-en-28-oic acid), a pentacyclic triterpenoid, is present in fruits such as *Vaccinium oxycoccos.* It has several biological effects such as protection against oxidative damage [38] and lipid oxidation [39].

#### 2.1.1. Phenolic Acids

The main representatives of phenolic acids in cranberries belong to cinnamic and benzoic acid derivatives. There are presented hydroxybenzoic acid derivatives such as gallic acid (3,4,5-trihydroxybenzoic acid), dihydroxybenzoic acids (vanilic), 2,3-dihydroxybenzoic, 2,4-dihydroxybenzoic acids, p-hydroxyphenylacetic, hydroxycinnamic (coumaric) acids such as *m*-coumaric and *p*-coumaric acids, caffeic (3,4-dihydroxycinnamic), and ferrulic (4-hydroxy-3-methoxycinnamic) acids [37,40]. However Tian et al. [41] identified in press cake from cranberry (*V. oxyccocos*) juice processing only two phenolic acids, 3-O-caffeoylquinic acid and caffeic acid.

Benzoic acid level in European cranberry is cultivar dependent which has been confirmed by a study by Česonienė et al. [28] in 13 berry cultivars, with values from 4.3 mg/L for “Amalva” to 32.12 mg/L for “Maima” cultivars, with an average of 17.5 mg/L. However, berry juices of *V. macrocarpon* cultivars were defined by higher benzoic acid amounts, from 19.37 (“Howes”) to 72.42 mg/L (“Searles”); cultivars “Franklin”, “Le Munyon”, “Searles”, and “Early Richard” were selected as the best according to the benzoic acid amounts.

Due to Stobnicka and Gniewosz [11], the fruits of *Vaccinium oxyccocos* contain phenolic acids in the total amount of 389.5 mg/100 g, individually benzoic acid in the content of 214.6 mg/100 g, *p*-coumaric acid 77 mg/100 g, chlorogenic acid 96.3 mg/100 g, caffeic acid 1.4 mg/100 g, and gentistic acid 0.3 mg/100 g.

Ehala et al. [15] compared the phenolic acids profile of small berries such as European cranberry, bilberry, cowberry, strawberry, black currant, and red currant. The results showed that European cranberries and cowberries reached the highest levels of *p*-coumaric acid (2.03 and 1.71 mg/100 g, respectively) which is the predominant acid of all mentioned berries. However, it is much less than in the previously referred study.

#### 2.1.2. Flavonoids

Generally, in *Vaccinium* genus and so in cranberry fruit, there are three classes of flavonoids such as flavonols, anthocyanins, and proanthocyanidins [14]. To the predominated flavonoids in cranberry fruit belong flavonols included myricetin-3-galactoside, myricetin-3-arabinofuranoside, quercetin-3-galactoside, quercetin-3-glucoside, quercetin-3-rhamnospyranoside, and quercetin-3-O-(6′′-p-benzoyl)-galactoside [42].

Ehala et al. [15] determined flavonoid profiles of European cranberry (*V. oxycoccos*), bilberry (*V. myrtillus*), cowberry (*V. vitis-idae*), black currant, and strawberry. The results proved that predominant flavonol present in the assayed berry crops was quercetin, with the highest level in bilberry (1.28 mg/100 g fw) and European cranberry (0.52 mg/100 g fw) fruits. But the detected amounts were much lower than in the study of Häkkinen et al. [43], who also found quercetin as the main flavonoid in 25 different berries. The highest content they evaluated in bog whortleberry (15.8 mg/100 g fw), lingonberry (7.4–14.6 mg/100 g), and cranberry (8.3–12.1 mg/100 g), followed by chokeberry, sweet rowan, rowanberry, sea buckthorn berry, and crowberry. The concentration of quercetin evaluated by Stobnicka and Gniewosz [11] in ethanolic extract of *Vaccinium oxyccocos* fruit reached up 15.4 mg/100 g.

Another important flavonoid, myricetin, was also detected in cranberry and other fruits such as black currant, crowberry, bog whortleberry, blueberry, and bilberry, in amounts from 1.4 to 14.2 mg/100 g fw [43]. In *V. oxyccocos*, Stobnicka and Gniewosz [11] determined the content of myricetin in the level of 8.4 mg/100 g. Further there was found isorhamnetin too, 2.1 mg/100 g. Flavonols represented 23 to 31% of the phenolics analyzed in *Vaccinium* species [43].

Taruscio et al. [37] evaluated the content and profile of flavonoids (by HPLC/ Diode-Array Detection (DAD-) /MS techniques) presented in nine *Vaccinium* species extracts, including *Vaccinium oxycoccos*. The results of an experiment showed that flavonoid fraction contained anthocyanidins, flavan-3-ols, and flavonol aglycons. The highest variation, detected among six cranberry varieties, was characterized for flavonol content (50–70%) (quercetin, kaempferol, and myricetin) that has been proved also by study of Bilyk and Sapers [44]. Differences were found also in the flavan-3-ol profile of three *Vaccinium* species (European cranberry, American cranberry, lingonberry) in an epicatechin/catechin ratio in American cranberry concentrates compared with the other berries [45].

The content of flavonoids could be affected not only by vegetational conditions but also by processing procedures such as drying technics that may influence their values. The effect of freeze and thermal drying on the flavonoid content in the fruits of European cranberry (*Oxycoccus palustris* Pers.) was studied by Adamczak et al. [46]. The results of the experiment showed a significant influence of drying conditions on flavonoids content. The level of flavonoids in the thermally-dried fruits was from 144 to 167 mg/100 g dw, which are higher values compared to freeze-dried samples (123–141 mg/100 g dw). The drying of European cranberry fruit at the temperatures 35–40 °C guarantees higher flavonoid content than by lyophilization drying. In the thermally-dried fruits of *Oxycoccus palustris* they found twice more flavonoids than in the commercial raw material of this species dried under similar conditions, as evaluated Bylka and Witkowska-Banaszczak [47]. According to Abascal et al. [48], freeze drying insufficiently stabilizes some groups of pharmacologically active compounds, such as phenolics and others.

#### 2.1.3. Anthocyanins

Generally, *Vaccinium* fruits belong to the most important food sources of anthocyanins of blue, red, and purple colors. The best representative of this group is bilberry fruit (*Vaccinium myrtillus* L.), that comprise phenolic compounds as anthocyanins in amounts up to 90%, an amount of 600 mg/100 g fw, with glycosides of cyanidin, delphinidin, malvidin, peonidin, and petunidin [49].

In cranberries, the amount of anthocyanins is much lower than in bilberries and significant genetic variability was found especially in the levels of total and individual anthocyanins (i.e., cyanidin-3-galactoside, cyanidin-3-glucoside, cyanidin-3-arabinoside, peonidin-3-galactoside, peonidin-3-glucoside, and peonidin-3-arabinoside) [16]. Juices of cranberry fruit cultivars could be distinguished by prevailing individual anthocyanins with thermostable galactoside and glucoside conjugates.

The clones of European cranberry (18) of Lithuanian origin, due to a study by Česonienė et al. [8], accumulate anthocyanins on the average 99 mg/100 g. The amount of anthocyanins in Lithuanian Žuvintas clones was in the range from 56 to 137 mg/100 g, and in Čepkeliai clones from 36 to 206 mg/100 g.

The content in American cranberries measured for eight cultivars reached about two-fold higher values than European cranberries, due to results of 13 the cultivar determinations. The amount of total anthocyanins in European cranberries ranged from 40.7 mg/100 g to 207.3 mg/100 g. The accumulation and the content of anthocyanins are also dependent on berries ripening [16], and therefore, their exact color could be quite meaningful. Studies of the localization of anthocyanins in the berries of European cranberry showed that the amounts of these compounds in the berry skin are 6 to 10 times higher than in the pulp [31].

However, due to the examined accumulation of anthocyanins in berries of different wild clones of European cranberry, there was confirmed a strong negative correlation between berry weight and the amount of anthocyanins [16].

The differences among anthocyanin content of *V. oxycoccos* and *V. macrocarpon* species studied Česonienė et al. [28], who compared total anthocyanins content in berry juice of nine American cranberry and thirteen European cranberry cultivars. Cultivars of *V. macrocarpon* accumulated on average 92.45 mg/L of total anthocyanins and therefore they are better sources of anthocyanins than European cranberry considering the average content that was approximately half of the amount (42.54 mg/L). Wang et al. [50] detected among large cranberry cultivars wide variability for anthocyanins content, averaging 25–65 mg/100 g of ripe fruit at harvest. As Tikuma et al. [19] determined the cultivar of *V. macrocarpon* Ait. “Early Black” contained the highest amount of anthocyanins (105 mg/100 g) in comparison to other cultivars. Also Latvian bred big cranberry cultivar “Septembra” showed a high level of anthocyanins (82.5 mg/100 g).

The highest anthocyanin content in juices from thirteen European cranberry cultivars (“Vaiva”, “Reda”, “Žuvinta”, “Vita”, “Amalva”, “Krasa Severa”, “Dar Kostromy”, “Sazonovskaja”, “Soontagana”, “Kuressoo”, “Nigula”, “Virussaare”, and “Maima”) was determined in “Sazonovskaja” and “Nigula” (93; 84.78 mg/L) cultivars, moderate amount contained cultivars “Amalva”, “Vaiva”, “Dar Kostromy”, “Kuressoo” (59.05–40.58 mg/L). “Vita”, “Maima”, and “Virussaare” cultivars were characterized by very low amount of anthocyanins in cranberry juice (28.19–12.29 mg/L) [28].

Andersen [51] proved that the anthocyanin pattern of European cranberry fruit is different from the mostly studied American cranberry varieties. In respect to anthocyanidins, peonidin-3-glucoside (41.9%) and cyanidin-3-glucoside (38.3%) represented the main fractions of anthocyanins isolated from fruits of *V. oxycoccus* L. Smaller amounts of 3-monoglucosides of delphinidin, petunidin, and malvidin and 3-monoarabinosides of peonidin and cyanidin were found, all anthocyanins together in the amount of 78 mg/100 g fw.

Generally, galactoside together with glucoside conjugates of cyanidin and petunidin comprised the largest percentage of total anthocyanins in the juices of *V. macrocarpon* and *V. oxycoccos* cultivars [14,16,28]. Quantitative HPLC-UV analysis revealed six anthocyanins in the berries of European cranberry, among which anthocyanin peonidin-3-galactoside dominated and comprised from 20.3 to 40.4% of the total anthocyanins in the juice. There were also detected cyanidin-3-galactoside (average 19.3%), cyanidin-3-glucoside (2.8%), cyanidin-3-arabinoside (20.2%), peonidin-3- galactoside (29.6%), peonidin-3-glucoside (8.1%), and peonidin-3-arabinoside (19.8%), whereas proportions of different compounds vary between the studied genotypes [16]. As the most abundant individual anthocyanins in freshly prepared juice from Finish cranberries (*V. oxycoccos*), cyanidin-3-arabinoside (23.1%), peonidin-3-galactoside (21.5%), cyanidin-3-galactoside (19.2%), and peonidin-3-arabinoside (14.1%) were also specified [52].

In the experiment by Brown et al. [53] and Česonienė et al. [28], the same six major anthocyanins were quantified in *V. oxycoccos* and *V. macrocarpon* berries. However, the ratio of glycosylated peonidins to cyanidins was about 20:80, as compared to 60:40 in *V. macrocarpon* [53]. Galactoside conjugates were the most prevalent anthocyanins and comprised 57.54% and 49.59%; arabinoside conjugates comprised 34.73% and 40.97%; and glucoside conjugates comprised 7.87% and 9.44% of TAC (total antioxidant capacity) in *V. macrocarpon* and *V. oxycoccos* berry juices, respectively. The most prevalent anthocyanins in both cranberry species are peonidin-3-galactoside (33.29 and 29.15%), cyanidin-3-galactoside (24.11 and 20.44%), and peonidin-3-arabinoside (16 and 19.64%) [28]. Similar amounts of prevailing anthocyanins were also examined by Vorsa and Polashock [54].

Depending on the type of product obtained in various processes, the amount of phenolic compounds of fresh, frozen, and freeze-dried cranberry fruit differ. Mazur and Borowska [35] showed that lyophilization of European cranberry fruit resulted in a seven-fold reduction in the content of anthocyanins, compared to the fresh fruit. In the fresh fruit, the content of cyanidin-3-glucoside was 58.3 mg/100 g of the product, while in frozen and freeze-dried fruits it was 39.8 and 55.2 mg/100 g, respectively. Due to study of Tian et al. [41], anthocyanins in the European cranberry press cake from juice processing, were represented by glycosides of cyanidin and peonidin, mainly as 3-O-galactoside, 3-O-glucoside, and 3-O-arabinoside.

Although anthocyanidins belong to the important antioxidants, Brown et al. [53] detected a strong negative correlation (*r* = − 0.92) between the anthocyanin content and the relative antioxidant potential. No linear dependence between total amount of anthocyanins and phenolics was found by Česonienė et al. [8]. The amount of anthocyanins was not the main factor, which determines total amount of polyphenols in the berries of European cranberry.

#### 2.1.4. Proanthocyanidins

Proanthocyanidins belong to the class of polyphenols with repeating catechin and epicatechin monomeric units. Proanthocyanidins are the leading compounds of the phenolic compounds of European cranberry [16]. The European cranberry accumulated 1.5–2.0 mg/100 g proanthocyanidins [33].

Catechin, epicatechin, and A-type dimers and trimers were found to be the terminal units of isolated proanthocyanidin fractions. Cranberry proanthocyanidins are primarily dimers, trimers, and larger oligomers of epicatechin [14]. European cranberries were noted to contain A-type dimers and trimers also by Määttä-Riihinen et al. [55]. Kylli et al. [36] tested European, small-fruited cranberries (*V. microcarpon*) and lingonberries (*V. vitis-idaea*) for their flavonoid profile. The results of experiments proved that the main phenolic compounds in them were proanthocyanidins comprising 63–71% of the total phenolic content. Proanthocyanidins were presented mainly by catechin, epicatechin, gallocatechin, and epigallocatechin units. Cranberry proanthocyanidins comprise a group of heterogeneous chemical structures, characterized by their constitutive units, types of linkage, and degree of polymerization. Proanthocyanidins of cranberry fruit are represented by dimers and trimers, oligomers, and polymers. Proanthocyanidins can be divided into three groups: dimers and trimers, oligomers (mDP (mean degree of polymerization) = 4–10), and polymers (mDP > 10). Catechin, epicatechin, and A-type dimers and trimers were found to be the terminal units of isolated proanthocyanidin fractions.

Jungfer et al. [56] found that three A-type trimers and procyanidin A2, identified as major bioactive compounds in *V. macrocarpon*, are present only in trace amounts in the European cranberry (*V. oxycoccus* L.), and at substantially higher amounts in lingonberry (*V. vitis-idaea* L.). According to the authors, the mentioned differences are responsible for different biological and clinical effect of berries, especially on the urinary tract. Differences can be used to prove the authenticity of compared species. But Boudesocque et al. [57] determined losses of proanthocyanidins A2 and B1 that may occur during manufacturing processes and storage of cranberry extracts.

Proanthocyanidins are responsible for organoleptic, anti-inflammatory, antibacterial, and antiviral properties of cranberry fruits [16].

## 3. Antioxidant Activity of Cranberry Fruit

Generally, the most important groups of bioactive compounds in European cranberry fruit are polyphenolic and triterpene compounds, displaying strong antioxidant properties and the ability to alleviate some chronic diseases [58].

Antioxidants are abundantly present in the genus *Vaccinium*, and numerous studies have been focused on their antioxidant activity [59,60,61]. Berries of all important *Vaccinium* species (*V. macrocarpon*, *V. oxycoccos* L., *V. vitis-idaea* L.) have been proved to possess a strong potential to prevent the free radical reactions from continuing [31,62]. Cranberry fruits inhibit oxidative processes including oxidation of low-density lipoproteins [63], and oxidative and inflammatory damage to the vascular endothelium [64].

Denev et al. [65] evaluated antioxidant properties of 26 Bulgarian fruits by ORAC (Oxygen radical absorbance capacity) method. From investigated fruits, cranberries (70 μmol TE.g^−1^ fw) showed the 10th best result after elderberry (205 μmol TE.g^−1^ fw), brier, chokeberry, hawthorn, blueberry, black currant, rowanberry, blackthorn, and blackberry. They found a good linear correlation between total polyphenol content and antioxidant capacity with R^2^ = 0.899.

Borowska et al. [31] compared wild cranberry (*V. oxycoccos*) fruit and five American cranberry cultivars (“Ben Lear”, “Bergman”, “Early Richard”, “Pilgrim”, and “Stevens”) in terms of their antioxidant properties measured as DPPH·, ·OH, and ABTS^+^ radical scavenging capacity. The results showed that widely grown European cranberry is characterized by high antioxidant activity (highest ABTS^+^ scavenging capacity of all cultivars, similar OH, and lower DPPH scavenging capacity). Statistically significant differences (*p* < 0.05) were observed between the wild cranberry and other analyzed cultivars.

Three *Vaccinium* fruits species (*V. oxycoccos* L., *V. vitis-idaea* L., and *V. macrocarpon* Aiton) due to their antioxidant potential studied Brown et al. [3]. An amount of cranberry tissue for 50% reduction in DPPH response was the lowest for *V. oxycoccos* berries, therefore European cranberry has better relative antioxidant potential than American cranberry or cowberry.

In the research of Ehala et al. [15], the antioxidant capacity of *Vaccinium oxycoccus, V. myrtillus*, and *V. vitis-idaea* species with the results 0.84, 1.89, 1.76 µM of ascorbic acid equivalent per 100 g of frozen berries was compared. Berries of bilberry and cowberry showed better results of antioxidant capacity than European cranberry. They studied also a possible relation to the total polyphenols content, with the amount for the berries 18.08, 43.43, and 35.95 mg of tannic acid equivalent per 100 g, respectively. The experiment outcomes proved that total phenolic level of *Vaccinium* species berries was correlated with their antioxidant activity.

The positive correlation between phenolic compounds and antioxidant activity of *Vaccinium* genus was confirmed also by experiments by Viskelis et al. [20], Seeram et al. [66], and Zheng and Wang [67]. The high values of antioxidant activities of lingonberry (*V. vitis-idaea L*.), cranberry (*V. oxycoccus L*.), and bog blueberry (*V. uliginosum* L.) seem to be related to their high content of catechin or proanthocyanidins, in comparison to other berry crops [33,55,67], while in the studies of Kähkönen et al. [24] and Brown et al. [3] an antioxidant activity of *Vaccinium oxycoccos* L., *V. vitis-idaea* L., and *V. macrocarpon* was not found to correlate with indolamine levels, and anthocyanin content was in a negative correlation with antioxidant activity. Vitamin C content positively correlated with an antioxidant activity of these berries. However, the presence of antioxidants and their amount in fruits, due to the presented results of mentioned studies, depends on genetic and environmental factors such as cultivar and variety, climate, place of origin, sun exposure, fertilization, harvest time, irrigation, etc. The concentrations of individual polyphenols during cranberry fruits ripening, as Oszmiański et al. [68] detected, were similar, but their overall values differed significantly. Immature fruits had the lowest level of polyphenols that increased in semi-mature fruits and did not change in mature cranberry fruits too much. The quantity of phytochemical compounds during cranberry fruit ripening depended on cultivar.

Due to the good antioxidant activity of European cranberry fruit, the extract could be utilized as an additive to meat products for inhibiting unfavorable storage changes of lipids to impede lipid oxidation [69]. The effect of cranberry juice on oxidative changes occurring in meat products determined also Tyburcy et al. [70]. Cranberry juice, in the amount of 5% of the meat weight, was added to the thermally processed pork burgers, which were stored for seven days at 3 to 7 °C, and juice was added also to a raw beef stuffing. A 5% addition of the cranberry juice caused decreasing of TBARS (thiobarbituric acid reactive substances) values, which are formed as a byproduct of lipid peroxidation of burgers, to twice or by three times the value of the control sample. As authors mentioned too, cranberry juice was a good color stabilizer of the raw beef stuffing.

## 4. Biological Activities of European Cranberry

*Vaccinium macrocarpon* is commercially utilized species and the subject of biological and clinical research, whereas there has been a limited number of studies on *Vaccinium oxycoccos*, especially in anticancer, cardioprotective or treatment of the urinary tract. The majority of published papers have been focused on antimicrobial effect of European cranberry.

### 4.1. Antiinflammatory Effect

Among the important biological activities of cranberries is also anti-inflammatory activity. Anti-inflammatory properties of cranberry fruit can be explained by a high level of quercetin [71] that decreases cytokine production in macrophages, reduces COX-2 mRNA expression, and inhibits TNF-*α*–dependent NF-*κ*B activation [72,73].

Kylli et al. [36] studied the mechanism of anti-inflammatory effect of small cranberry and lingonberry extracts. The results of experiment showed that cranberry (*V. microcarpon*) phenolic extract inhibited LPS (lipopolysaccharide) induced NO (nitric oxide) production in a dose-dependent manner, but it had no major effect on iNOS of COX-2 expression. At a concentration of 100 μg/mL cranberry phenolic extract inhibited LPS-induced IL (interleukin)-6, IL-1β, and TNF-α production (tumor necrosis factor). Lingonberry phenolics had no significant effect on IL-1β production but inhibited IL-6 and TNF-α production at a concentration of 100 μg/mL similar to cranberry phenolic extract.

### 4.2. Antimicrobial and Antiviral Activity of Cranberry Fruit

Plant materials, in general, are often rich in various secondary metabolites that are important as a natural defense mechanism for living organisms and are known to have antimicrobial properties in vitro. Natural antimicrobial compounds could be effective against selected bacteria and fungi components such as flavonoids, e.g., quercetin [74,75]. Before bacterial infection, bacterial adhesion to the cell surface is crucial. Berries from the *Vaccinium* species represent a possible source of anti-adhesives against bacterial infections [76,77].

The antimicrobial effects of American cranberry concentrates against bacterial pathogens (*Staphylococcus aureus*, *E. coli* O157:H7) are well known [78,79]. Rauha et al. [10] proved this fact also for *Vaccinium oxyccocos* fruits with a methanolic extract isolated from Finnish berries. They demonstrated an effectiveness of the berry extract against bacterial strains. Moderate activity has been shown against *Staphylococcus aureus* and clear antimicrobial activity against *Escherichia coli*, an important Gram-negative bacterium. The berry extract failed to inhibit *Staphylococcus epidermidis*, *Bacillus subtilis*, *Micrococcus luteus*, and the mold *Aspergillus niger*, and also the growth of the yeast *Candida albicans*. Also, Česonienė et al. [27] determined the antimicrobial properties of different wild clones of European cranberry by the agar well diffusion method against these bacteria. European cranberry extracts inhibited the growth of a wide range of human pathogenic bacteria, both Gram-negative (*Escherichia coli* and *Salmonella typhimurium*) and Gram-positive (*Enterococcus faecalis*, *Listeria monocytogenes, Staphylococcus aureus*, and *Bacillus subtilis*).

Moreover, berry juice of *V. oxycoccus* displayed binding activity of *Streptococcus agalactiae* and *Streptococcus pneumoniae* [77]. It has been determined that *S. pneumoniae* has binding activity to low molecular size fractions of cranberry (*V. oxycoccos* L.) and bilberry (*V. myrtillus* L.) juices in a microtiter well assay.

Antibacterial inhibitory activity of European cranberry, as Hellström [80] evaluated, is given by polyphenolic subfraction at a concentration of 5 mg/100 ml. The activity can be explained by high level of polyphenols, especially proanthocyanidins, about 400 mg/100 g. Proanthocyanidins are known to prevent the adhesion of several bacteria. A-type dimers and trimers have been found in European cranberries and in a good amount in lingonberries and American cranberries [14]. Similarly Kylli et al. [36] also proved that proanthocyanidins are responsible for antimicrobial activity of *V. microcarpon*. Polymeric proanthocyanidin fraction of cranberries displayed a strong antimicrobial effect against *Staphylococcus aureus*; no effect was determined on other bacterial strains (*S. enterica sv. Typhimurium, Lactobacillus rhamnosus*, and *Escherichia coli*).

In an experiment by Ermis et al. [45], there was shown a possibility to inhibit the growth of visible colonies of several fungi with concentrate of cranberry in fruit spreads (raspberry–aloe vera; strawberry–lime) with reduced sugar, which is a main reason for a growth of microorganisms in low-calorie jams. The antifungal activities of cranberry concentrate were studied in vitro against selected fungi *Absidia glauca, Penicillium brevicompactum, Saccharomyces cerevisiae*, and *Zygosaccharomyces bailii*. The concentrate was able to inhibit growth of visible colonies of most xerophilic and non-xerophilic fungi. For both fruit spreads with cranberry concentrate *A. glauca* was not able to grow, the growth of *P. brevicompactum* on the spread was inhibited at 3% cranberry concentrate, and *S. cerevisiae* could not grow at a concentration of 18%. *Z. bailii* was the most resistant fungus, the highest concentration (24%) was able to inhibit its growth by 29.8% only for raspberry–Aloe vera spread.

Extract from European cranberry also represents an interesting candidate as a natural preservative of minced pork meat. Water, ethanol fruit, and pomace extracts were tested due to their antimicrobial activity as the growth inhibitors of *S. aureus*, *Listeria monocytogenes*, *Salmonella enteritidis*, and *E. coli* in inoculated fresh minced pork meat containing 2.5% extract. Extracts inhibited Gram-positive bacteria strains stronger than Gram-negative, but did not display antifungal activity. Water–ethanol fruit and pomace extracts displayed more effective antibacterial properties than ethanolic and aqueous fruit and pomace extracts [11]. Cranberry pomace extracts contained stilbenes (resveratrol) and more organics acids and flavonols than fruit extracts, both contained also terpenes in ethanol extract (ursolic acid).

Wild cranberry (*V. oxycoccos* L.) juice fraction, and also bilberry (*V. myrtillus* L.), lingonberry (*V. vitis-idaea* L.), and crowberry (*Empetrum nigrum* and *E. hermaphroditum* L.) were studied as antimicrobial agents against bacterium *Neisseria meningitides* using a microtiter broth microdilution assay. This bacterium infects human mucosal cell surfaces and colonizes the nasopharyngeal epithelium, is transmitted from person to person, and causes meningitis. The berry juice molecular size fractions of 10–100 kDa inhibited the binding of isolated *N. meningitidis* pili to membrane-bound epithelial cells in a dot assay. Toivanen et al. [76] proved that polyphenolic fractions containing anthocyanins and proanthocyanidins displayed antiadhesion activity against this human pathogen, and thus *Vaccinium* berries could be promising sources against meningococcal adherence. The most effective adhesion inhibition of 75% was achieved with cranberry juice polyphenolic fraction followed by crowberry (63%), bilberry (63%), and lingonberry (57%) juice phenolic fractions.

Huttunen et al. [81] tested fractions of *V. oxyccocos* juice against pneumococcal binding in human bronchial cells (Calu-3). The antiadhesion activity was achieved at a concentration of 8.7  mg/g of soluble solids, which contain small amounts of polyphenols. The antimicrobial activity of the studied berry juice fractions was found to be remarkable; pneumococcal growth was inhibited totally at a concentration of about 86  mg/g.

As was pointed out before, cranberry fruit extracts possess, besides antibacterial [82] and antifungal effect, also antiviral activity. Berries belonging to the genus *Vaccinium*—blueberry, Natsuhaze (*V. oldhamii*), bilberry, and European cranberry were compared due to the anti-influenza viral effects [83]. As the authors proved, cranberries belong to the species with the high antiviral effect, comparable to that of bilberry, Natsuhaze, and blackcurrant, while blueberries (Rabbiteye varieties) had the strongest effect. A positive relationship was observed between anti-influenza viral activity and total polyphenol content, which indicates the possibility of a high content of polyphenols as one of the most important factors in the antiviral effects of berries.

Summarization of the main bioactive compounds (Table 3) of European cranberry with biological activities, which have been proved by several studies in in vitro and other models, and is presented in Table 4.

### 4.3. Urinary Tract Protection

The numerous studies proved that species of the genus *Vaccinium* can be utilized in the treatment of several health problems such as urinary tract infections, e.g., American cranberries [99,100,101,102], as cranberry juice is the most studied means considered as greatly important for preventing urinary infections in high-risk populations [89,90]. Otherwise, also European cranberry (*V. oxycoccos)*, lingonberry, and blueberry contain bioactive compounds effective against *E. coli* but their evidence in urinary infection prevention is still in doubt and not exactly clear [93,94,103]. The positive effects of cranberry extract have been correlated to A-type linkage proanthocyanidins. In vivo studies proved that cranberry proanthocyanidins (190 μg/ml) have a relationship to adhesion, motility, biofilm formation, and iron and stress response of uropathogenic *Escherichia coli.* Furthermore, cranberry proanthocyanidins influence the transcriptional profiles of Escherichia coli, anti-adhesion effect is mainly the effect of proanthocyanidins on “strategic” genes involved in *E. coli* adherence [85].

Commercial cranberry products for urinary infections treatment are produced as monopreparations or the mixtures of American cranberry, European cranberry, and/or lingonberry. They are defined by different proanthocyanidins pattern. A-type dimers and trimers reached up the highest content in lingonberry followed by American cranberry, the lowest level was detected in the European cranberry (*V. oxycoccos*). All three species contain A-type dimers of proanthocyanidins that are the most important in the anti-adherent activity. These compounds are considered as well effective in the treatment of urinary infections. However, there are remarkable differences in the procyanidin profiles and concentrations, especially the lack of A-type trimers in *V. oxycoccos*. Therefore, the effectiveness against urinary infections may be variable among the *Vaccinium* species [56].

Studies of Kontiokari et al. [91,92] pointed to the effectiveness of berry juices from a mixture of cranberries (*V. oxycoccos*) and lingonberries as well as cloudberry juice against urinary tract infection. According to research by Abascal and Yarnell [104], *V. oxycoccos* berries are more effective against cystitis.

Generally, cranberries exhibit a dose-dependent inhibition of the adherence of *E. coli* to uroepithelial cells. A-type proanthocyanidins play an important role in the mechanism of this inhibition [90,104,105,106]. Cranberry-derived compounds such as A-type proanthocyanidins in synergism with another polyphenols interfere with adhesion of bacteria (including multi-drug-resistant *E. coli*) to epithelial cells of the urinary tract and suppress inflammatory cascades [86,87]. The research of Kylii et al. [36] pointed out that although polymeric proanthocyanidin extracts of cranberries had no effect on *Escherichia coli*, oligomeric and polymeric fraction of cranberries showed an inhibitory effect on hemagglutination of *E. coli*, which expresses the M hemagglutinin.

According to Hidalgo et al. [107], more research is needed for the determination of co-active compounds that are helpful in anti-adherence activity, especially anthocyanins with anti-inflammatory activity. There is also no clear-cut evidence that the consumption of cranberry juice products prevent urinary tract infections caused by *E. coli* [108]. The results of meta–analyses (24 studies with 4473 participants) showed that effectiveness of cranberry products (juices, tablets) extracted from *V. oxyccocos*, *V. macrocarpon*, and *V. vitis-idaea* were not significantly different to results with antibiotic treatment for women and children [95,96].

### 4.4. Cardioprotective Effect

The regular consumption of cranberry fruit have a positive effect on hypertension, inflammation, oxidative stress, endothelial dysfunction, arterial stiffness, and platelet function. Polyphenols in cranberry reduce ROS (reactive oxygene species), decrease concentration of inflammatory cytokines, and enhance endothelium—dependent vasodilation and inhibited platelet activation [109].

The anti-inflammatory effect of European cranberry could have a positive effect on blood pressure and vascular function. Kivimäki et al. [26,97] determined the effect of eight weeks of treatment by Finnish berry juices, European cranberry, and lingonberry on blood pressure and vascular function of spontaneously hypertensive rats. But only the treatment with lingonberry juice mostly normalized the impaired endothelium-dependent relaxation in comparison with rats fed by cranberry juice and control rats. In the arteries of lingonberry-treated rats, the relaxation was partly due to NO, but also dependent on EDHF (endothelium-derived hyperpolarization factor). European cranberry and lingonberry cold-compressed juices in long-term treatment of hypertensive rats showed changes in expression of anti-inflammatory and anti-thrombotic mediators in vasculature that can explain the mechanism of improved endothelial function. The mRNA expressions of angiotensin-converting enzyme 1 (ACE1), cyclooxygenase-2 (COX2), monocyte chemoattractant protein 1 (MCP1), and P-selectin were significantly reduced in the cranberry and lingonberry groups.

Cranberries could be effective also in the prevention of heart diseases and ulcer illnesses of the digestive system [110]. However, as Kalt et al. [88] pointed out, anti-adhesion and anti-platelet bioactivities do not correlate directly with total phenolics, anthocyanins, or proanthocyanidin content, and the beneficial effect of fruit phenolics can be realized only after their digestion and absorption in the body.

### 4.5. Anticancer Effect

Because of the high antioxidant activity of Vaccinium species, especially due to anthocyanins content, cranberry is able to inhibit the oxidative process related to tumorigenis. Furthemore, the in vitro model experiment showed direct antiproliferative or growth inhibitory properties of the in vitro model [14].

In *V. oxycoccos* fruits there is presented ursolic acid that inhibits UVA-radiation-induced oxidative damage in human keratinocytes [38] and offers a remarkable protection against UVB-induced lipid peroxidation, oxidative stress, and DNA damage [39].

Berry fruits such as blueberries, strawberries, raspberries, and cranberries inhibit multiple stages of carcinogenesis, inhibits the growth of human oral (KB, CAL-27), breast (MCF-7), colon (HT-29, HCT116), and prostate (LNCaP) tumor cell lines. With increasing concentration of berry extract (from 25 up to 200 µg/mL) they detected increasing inhibition of cell proliferation in all of the cell lines, with different degrees of potency between cell lines [93].

Some phytochemicals, contained in fruits of the *Vaccinium* genus, are expected to affect cancer-related processes. Proanthocyanidins and flavonoids, presented in cranberries and other *Vaccinium* berries, show some promising effects toward limiting processes involved in tumor invasion and metastasis. The fruits of *V. oxycoccos* are able to suppress the proliferation of human breast cancer MCF-7 cells which can be attributed to the initiation of apoptosis and the G1 phase arrest too [84].

Unfortunately, cranberry-based preparations (i.e., tablets, capsules) and juice available in European market are most often originated from *V. macrocarpon*, and the fruit of *V. oxycoccos* was used very rarely [111].

## 5. Conclusions

Although it has a wide range of biologicaly active substances, the European cranberry (*Vaccinium oxycoccos*), a lesser known type of fruit, is still underutilized. In the same way as the large cranberry, the European cranberry also represents an excellent source of bioactive compounds, especially polyphenolic compounds (i.e., flavonoids, anthocyanins, and phenolic acids). On the other hand, the geographical distribution of European cranberry is wider (in natural bogs of Europe, Asia, and North America) and it is less demanding in comparison with large cranberry. The consumption of European cranberry fruits and their products such as juice drinks, jams, jellies, and sauces is beneficial especially due to its antioxidant properties. European cranberry represents important natural preservatives against bacterial and fungal growth. Also, their anti–inflammatory properties can be helpful in the prevention and treatment of cardiovascular problems and several types of cancer diseases. Taking into account various beneficial effects of small cranberries on human health, also in folk medicine, the consumption of these fruits and their products is widely recommended.

## Figures and Tables

**Table 1:** The overview of major phenolic compounds in European cranberry.

Phenolic Compounds		Content (mg/100 g fw or as Mentioned in the Brackets)	References
Phenolic acids	Benzoic acid	99.6–214.6	Stobnicka and Gniewosz [11]
	*p*-coumaric acid	2.0–78.0	Stobnicka and Gniewosz [11], Ehala et al. [15]
	Chlorogenic acid	61.0–96.3 7.8% (% of all phenolic acids)	Stobnicka and Gniewosz [11] Häkkinen et al. [25]
	Caffeic acid	0.7–1.4 12.2% (% of all phenolic acids)	Stobnicka and Gniewosz [11] Häkkinen et al. [25]
	Ferrulic acid	68.1% (% of all phenolic acids)	Häkkinen et al. [25]
Anthocyanins	Anthocyanins	12.4–207.3	Kivimäki et al. [26], Česonienė et al. [27]
	Cyanidin-3-galactoside	13.1–26.8% (mean 19.8% of all anthocyanins) 19.3% (% of all anthocyanins) 20.4% (% of all anthocyanins)	Česonienė et al. [27] Česonienė et al. [16] Česonienė et al. [28]
	Cyanidin-3-glucoside	0.09–13.4% (mean 3.4% of all anthocyanins) 2.8% (% of all anthocyanins) 3.2% (% of all anthocyanins)	Česonienė et al. [27] Česonienė et al. [16] Česonienė et al. [28]
	Cyanidin-3-arabinoside	16.5–40.5% (mean 21.7% of all anthocyanins) 20.2% (% of all anthocyanins) 21.3% (% of all anthocyanins)	Česonienė et al. [27] Česonienė et al. [16] Česonienė et al. [28]
	Peonidin-3-galactoside	5.9–42.8% (mean 30% of all anthocyanins) 29.6% (% of all anthocyanins) 29.2% (% of all anthocyanins)	Česonienė et al. [27] Česonienė et al. [16] Česonienė et al. [28]
	Peonidin-3-glucoside	1.4–23.3% (mean 7.4% of all anthocyanins) 8.1% (% of all anthocyanins) 6.2% (% of all anthocyanins)	Česonienė et al. [27] Česonienė et al. [16] Česonienė et al. [28]
	Peonidin-3-arabinoside	3.4–28.5% (mean 17.4% of all anthocyanins) 19.8% (% of all anthocyanins) 19.6% (% of all anthocyanins)	Česonienė et al. [27] Česonienė et al. [16] Česonienė et al. [28]
Flavonoids	Quercetin	0.52–15.4 79.9% (% of all flavonoids)	Ehala et al. [15], Stobnicka and Gniewosz [11], Häkkinen et al. [25]
	Myricetin	8.4–11.2 18.2% (% of all flavonoids)	Stobnicka and Gniewosz [11], Häkkinen et al. [25]
	Epicatechin	3.1–6.3	Stobnicka and Gniewosz [11]
Proanthocyanins		1.5–5.3	Kivimäki et al. [26], Koponen et al. [29], Ogawa et al. [30]

**Table 2:** Comparison of the polyphenolic spectrum of *V. oxycoccos* and *V. macrocarpon* fruit and pomace ethanol extracts [11,32].

Phenolic Compounds	Content (mg/100 g fw)		
	*V. oxycoccos* Fruit Extract	*V. oxycoccos* Pomace Extract	*V. macrocarpon* Pomace Extract
Benzoic acid	214.6	115.0	256.9
*p*-coumaric acid	77.0	175.0	184.3
Chlorogenic acid	96.3	408.7	656.9
Caffeic acid	1.4	36.5	31.2
**Sum of acids**	389.5	777.0	1173.8
Quercetin	15.4	25.2	11.5
Epicatechin	6.3	5.7	12
Isorhamnetin	3.5	1.5	0.9
**Sum of flavonols**	36.3	81.5	42.9

**Table 3:** The major bioactive compounds of European cranberry fruit and their effect.

Bioactive Compounds	Biological Effect	References
Quercetin	anti-inflammatory	Mlcek et al. [71], Liu et al. [72], Kim et al. [73]
	antibacterial and antifungal	Cushnie et al. [74,75]
Proanthocyanidins	anticancer	Masoudi et al. [84]
	antimicrobial	Neto et al. [14], Kylli et al. [36]
	urinary tract protection	Jungfer et al. [56], Ranfaing et al. [85], Gupta et al. [86], Vasileiou et al. [87]
	cardioprotective	Kalt et al. [88]
Resveratrol	antibacterial, antifungal	Stobnicka et al. [11]
Anthocyanins	antibacterial	Toivanen et al. [76]
	cardioprotective	Kalt et al. [88]

**Table 4:** Summarization of the evidence of European cranberry biological activities.

Effect	Studied Models	Mechanism of Action	References
**Antibacterial and antifungal activities**	agar well diffusion method; human epithelial cells	antiadhesion activity (blocking bacterial adhesion) against *Neisseria meningitidis, Streptococcus agalactiae, Streptococcus pneumoniae*	Toivanen et al. [76], Toivanen et al. [77]
	in vitro studies (minced pork meat)	inhibition of the growth of *Escherichia coli*, *Salmonella Enteritidis*, *Listeria monocytogenes*, *Staphylococcus aureus*	Stobnicka and Gniewosz [11]
	agar well diffusion method	inhibitory effect on hemagglutination of *E. coli*; the growth inhibition of *Salmonella typhimurium*, *Enterococcus faecalis*, *Listeria monocytogenes*, *Bacillus subtilis*	Česonienė et al. [27], Kylli et al. [36]
	in vitro studies (sugar reduced fruit spreads)	inhibition of growth of *Absidia glauca*, *Penicillium brevicompactum*, *Saccharomyces cerevisiae* and *Zygosaccharomyces bailii*	Ermis et al. [45]
	diffusion methods; human bronchial cells (Calu-3)	Antibacterial inhibitory activity against *Staphylococcus aureus*, *Escherichia coli,;* blocking bacterial adhesion against pneumococcal binding of *Streptococcus pneumoniae*	Rauha et al. [10], Huttunen et al. [81]
**Prevention of urinary tract infections (UTI)**	in vitro studies	effect of type-A proanthocyanidins; inhibition of the adherence of *E. coli* to uroepithelial cells	Davidson et al. [89], Shamseer and Vohra [90]
	women participants; meta-analyses; in vitro studies,	prevention of UTI, blocking of fimbrial adhesion of causative bacterium *E. coli* to colonise the uroepithelial cells	Kontiokari et al. [91], Kontiokari et al. [92], Jepson et al. [93], Jepson and Craig [94], Jepson et al. [95], Liska et al. [96], Ranfaing et al. [85]
**Cardioprotective effect**	in vitro model, rats fed with juice	vascular anti–inflammatory properties, inhibition of LPS (Lipopolysaccharide-) induced NO (nitric oxide) production, inhibition LPS-induced IL-6, IL-1β and TNF-α production	Kylli et al. [36], Kivimäki et al. [26]
	Spontaneously hypertensive rats (SHR) model	normalization of the impaired endothelium-dependent relaxation of mesenteric arteries, activity of endothelium-derived hyperpolarizing factor	Kivimäki et al. [97]
**Anticancer activity**	in vitro model (human oral, breast, colon tumor cells)	inhibition of stages of carcinogenesis, stimulation of the apoptosis of cancer cells	Seeram et al. [98], Masoudi and Saiedi [84]
	human prostate cancer cells	inhibition of specific temporal NMP (1-Methyl-2-pyrrolidone) regulators	Seeram et al. [98]
